# Tanshinone IIA Accomplished Protection against Radiation-Induced Cardiomyocyte Injury by Regulating the p38/p53 Pathway

**DOI:** 10.1155/2022/1478181

**Published:** 2022-08-22

**Authors:** Gang Wang, Li Ma, Bowen Wang, Fentang Gao, Jianfeng Li, Hongyi Cai, Juan Wang, Tiancheng Zhang, Hao Guo, Ping Xie, Yi Li

**Affiliations:** ^1^The First Clinical Medical College, Gansu University of Chinese Medicine, Lanzhou 730000, China; ^2^Gansu Provincial Maternity and Child-Care Hospital, Lanzhou 730000, China; ^3^Department of Cardiovascular Medicine, Gansu Provincial Hospital, Lanzhou 730000, China; ^4^Department of Radiotherapy, Gansu Provincial Hospital, Lanzhou 730000, China; ^5^School of Medicine, Xiamen University, Xiamen 361000, China; ^6^School of Stomatology, Lanzhou University, Lanzhou 730000, China

## Abstract

**Background:**

Radiotherapy is one of the major strategies for treating tumors, and it inevitably causes damage to relevant tissues and organs during treatment. Radiation-induced heart disease (RIHD) refers to radiation-induced cardiovascular adverse effects caused by thoracic radiotherapy. Currently, there is no uniform standard in the treatment of RIHD.

**Methods:**

In our group study, by administering a dose of 4 Gy radiation, we established a radiation injured cardiomyocyte model and explored the regulatory relationship between tanshinone IIA and p38 MAPK in cardiomyocyte injury. We assessed cell damage and proliferation using clonogenic assay and lactate dehydrogenase (LDH) release assay. The measures of antioxidant activity and oxidative stress were conducted using superoxide dismutase (SOD) and reactive oxygen species (ROS). The apoptosis rate and the relative expression of apoptotic proteins were conducted using flow cytometry and western blot. To assess p38 and p53 expressions and phosphorylation levels, western blot was performed.

**Results:**

Experimental results suggested that tanshinone IIA restored cell proliferation in radiation-induced cardiomyocyte injury (∗∗*P* < 0.01), and the level of LDH release decreased (∗*P* < 0.05). Meanwhile, tanshinone IIA could decrease the ROS generation induced by radiation (∗∗*P* < 0.01) and upregulate the SOD level (∗∗*P* < 0.01). Again, tanshinone IIA reduced radiation-induced cardiomyocyte apoptosis (∗∗*P* < 0.01). Finally, tanshinone IIA downregulated radiation-induced p38/p53 overexpression (∗∗∗*P* < 0.001).

**Conclusions:**

The treatment effects of tanshinone IIA against radiation-induced myocardial injury may be through the regulation of the p38/p53 pathway.

## 1. Introduction

Most tumor patients receive radiotherapy as a treatment for their tumors, and over half of them receive radiotherapy for their tumors [1]. Although the therapeutic effect of radiotherapy on tumors is effective, the damage of normal tissues or organs by radiation has attracted much attention [2]. Thoracic radiotherapy causes damage to the myocardium tissue, heart valves, and coronary arteries, collectively referred to as RIHD [3, 4]. The key injury mechanisms of RIHD from acute to chronic remain unanswered now [5]. Currently, there are barely options regarding the approach to the treatment of RIHD, and there are no specific drugs to prevent or treat RIHD. Therefore, the prevention and management of RIHD has become an issue to be addressed clinically, as RIHD can worsen the outcomes of cancer patients as well as the economic burden [6, 7].

Studies have found that ROS generated after radiation induce the destruction of intracellular macromolecules, including lipid peroxidation, enzyme inactivation with the interaction of DNA repair enzymes, and transcription factors. Damage to cells and intracellular macromolecules may lead to inflammatory responses, stress or apoptosis and necrosis, and activation of physiological mechanisms such as autophagy [4, 8, 9]. Therefore, reducing ROS production and inflammatory response is very important for treating radiation injury [10]. According to a previous study from our group, radiation causes increased apoptosis, growth inhibition, ROS secretion, p38 MAPK, and its phosphorylation in cardiomyocytes and cardiac fibroblasts, and this effect increases with radiation dose [11, 12]. P38, the most important member of the MAPK family in controlling inflammatory responses, is widespread in cells of various tissues and very important in the regulation of the oxidative stress, inflammatory responses, and apoptosis. It has been shown that p38 MAPK signaling pathway can degrade Bcl-2 and activate Bax, thereby playing a prime role in the process of apoptosis [13]. However, the p38 MAPK signaling pathway in radiation-induced cardiac injury remains unknown.

Tanshinone IIA is the main active component of Danshe. Studies have shown that tanshinone IIA has multiple biological activities, especially in antioxidative stress and attenuating inflammatory responses, and is used to treatment of heart diseases. It can improve and regulate myocardial metabolic dysfunction induced by hypoxia [14]. In addition, a protective effect of tanshinone IIA is also seen in myocardial ischemia; it could also inhibit proliferation of smooth muscle in vascular vessels and vascular intimal hyperplasia and inhibits cardiomyocyte Ca^2+^ influx [15]. According to our findings, tanshinone IIA could resist the negative effects of radiation on cardiomyocytes and cardiac fibroblasts, reduce the secretion of ROS, decrease the apoptosis of cells, and restore cell viability, thereby protecting cardiomyocytes [11, 12]. But there is no further research about the relationship between tanshinone IIA and p38 MAPK signaling pathway.

So we will continue to further investigate the role of p38/p53 signaling in RIHD by establishing a cardiomyocyte radiation injury model and the regulatory relationship between tanshinone IIA and p38/p53 pathway.

## 2. Materials and Methods

### 2.1. Culture, Radiation-Induced Injury Model, and Grouping

The H9c2 cell line was acquired from the Chinese Academy of Science (Shanghai, China). In an incubator at 37°C, 95% of the atmosphere and 5% CO_2_ were used to culture the cells. DMEM (Basal Media, Shanghai, China), 10% fetal bovine serum (ABWbio, Uruguay), and double antibiotic were used to prepare the complete culture medium. When the cell number reached 80-90% culture area, the cells were used for experiments.

To simulate radiation-induced myocardial injury, H9c2 cells were irradiated with a dose of 4 Gy using the Siemens Primus high-energy linear accelerator (Siemens AG, Erlangen, Germany). For the selection of radiation dose, we referred to the results of previous studies by our group [11, 12]. After irradiation, the complete medium was replaced with tanshinone IIA (final concentration, 10 *μ*g/mL, Shanghai First Chemical Company, Shanghai, China) and p38 MAPK agonist, anisomycin (final concentration, 4 *μ*g/mL, Cat. IA0770, Solarbio, Beijing, China). After 48 h, the cells were used in the experiment without prior change of medium.

The experimental groups were as follows: control group, 4 Gy group, 4 Gy+agonist group, 4 Gy+tanshinone IIA group, and 4 Gy+tanshinone IIA+agonist group.

### 2.2. Clonogenic Assay

The cells were digested with trypsin to prepare the cell suspensions and counted. 1000 cells were seeded in each 60 mm dish. After 24 h of cell adhesion, the radiation cell model was generated, and drug treatment was performed, and then, the cells were continued to be cultured for two weeks as per routine protocol. Two weeks later, after discarding old medium, the cells were fixed using methanol before staining with Giemsa staining solution (Cat. DM0002, Beijing Leagene Biotech). Finally, the number of cell colonies (≥50 cells) was counted, and the clone formation rate was calculated.

### 2.3. Cell Apoptosis Assay

We chose to evaluate apoptosis using the BD apoptosis detection kit (Cat. 559763, BD Pharmingen), and the experiments were performed exactly as per the instructions for use. A dose of 4 Gy was given to irradiate the cells and do the drug treatment; it was used to do experiments after 48 hours of routine culture. After making a cell suspension, 5 *μ*L each of PE and 7-AAD reagents were added, followed by a 15 min reaction protected from light. Finally, the results were detected by flow cytometer (BD LSRFortessa) within 1 h.

### 2.4. LDH and SOD Assay and ROS Flow Cytometric Analysis

Cells were given an X-ray irradiation dose of 4 Gy and given drug treatment and then routinely cultured for 48 h before use in experiments. Next, proteins were extracted from the cells. SOD activity and LDH release amount were detected with kits. Cells for experiments were treated with 0.1 mmol·L^–1^ DCFH-DA. DCFH-DA can be oxidized by ROS to fluorescent DCFH. Then, ROS levels were measured by flow cytometry. We used relevant kits to accomplish each of the above assays (Nanjing Jiancheng Bioengineering Institute, Nanjing, China). Positive control and negative control were added to the grouping according to the instruction of the ROS kit. The specific groupings in the ROS experiment were as follows: negative control, positive control, control group, 4 Gy group, 4 Gy+agonist group, 4 Gy+tanshinone IIA group, and 4 Gy+tanshinone IIA+agonist group.

### 2.5. Western Blot Analysis

First, lysis buffer and phenylmethylsulfonyl were used to extract proteins from the cells of each group. A polyvinylidene difluoride membrane was used to capture the proteins separated by electrophoresis (SDS-PAGE). Following blocking with 5% skim milk, primary antibodies (rabbit) were incubated on the membrane. A secondary antibody (goat anti-rabbit) was then incubated at room temperature with the membrane. Finally, the membrane was visualized on an imaging system (ChemiScope 6100, Clinx Science Instruments Co., Ltd.). Rabbit antibodies for GAPDH (Cat. 10494-1-AP), Bax (Cat. 50599-2-Ig), Bcl-2 (Cat. 26593-1-AP), p38 (Cat. 14064-1-AP), p53 (Cat. 10442-1-AP), and Caspase-3 (Cat. 19677-1-AP) were purchased from the Proteintech Group (Wuhan, Hubei, China). And rabbit antibodies for p-p38 (Cat. 9211) and p-p53 (Cat. 2521) were purchased from the Cell Signaling Technology (Danvers, Massachusetts, USA). We added the tanshinone IIA group and p38 agonist group to the grouping, in order to determine the independent effects of tanshinone IIA and agonist on cardiomyocytes. The specific groupings were as follows: control group, tanshinone IIA group, agonist group, 4 Gy group, 4 Gy+agonist group, 4 Gy+tanshinone IIA group, and 4 Gy+tanshinone IIA+agonist group. To determine the gray values of the banding patterns, we used Image J (version 1.53a), and the resulting gray values were then normalized. GAPDH was used as reference.

### 2.6. Statistical Analysis

These results were analyzed using SPSS 20.0 (IBM Corp) and presented as mean + SD. In contrasts between two groups, one-way analysis of variance (ANOVA) was used. It was considered statistically significant when *P* < 0.05 was used.

## 3. Results

### 3.1. Tanshinone IIA Abolished Radiation-Induced Inhibition of Cardiomyocyte Proliferation

**Figure 1:**
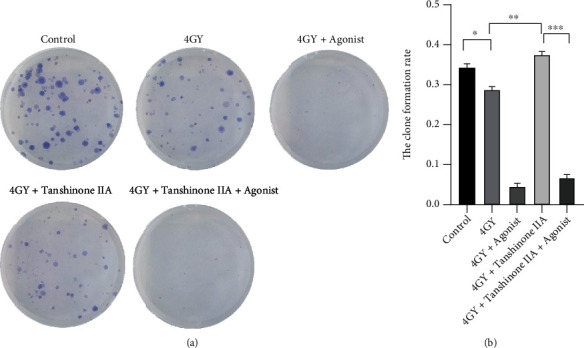
Effects of radiation and tanshinone IIA on cardiomyocyte proliferation. (a) The results of clonogenic assay suggested that radiation could inhibit normal cardiomyocyte growth, while tanshinone IIA could eliminate this inhibition to some extent. (b) The results of cell cloning experiments were expressed as the number of colonies of the cells. ^∗∗∗^*P* < 0.001, ^∗∗^*P* < 0.01, and ^∗^*P* < 0.05.

**Figure 2:**
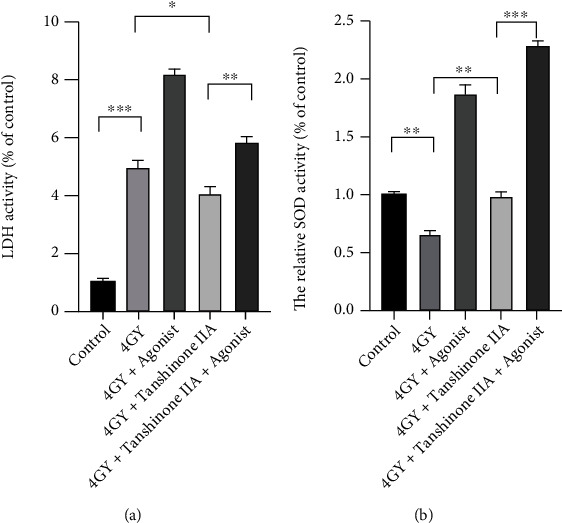
Radiation and tanshinone IIA effects on cardiomyocytes were detected by measuring LDH release (a) and SOD activity (b). The means ± SD are presented for each result (*n* = 3). ^∗∗∗^*P* < 0.001, ^∗∗^*P* < 0.01, and ^∗^*P* < 0.05. LDH: lactate dehydrogenase; SOD: superoxide dismutase.

Proliferation and damage of H9C2 cells were assessed by clonogenic assay and LDH release assay, and the results are shown in Figures 1 and 2(a). Comparing the 4 Gy group to the control group, the H9C2 cell clonality rate decreased (∗*P* < 0.05) while the LDH release level increased (∗∗∗*P* < 0.001). When found after treatment with tanshinone IIA, the cell clonality rate of H9C2 cells rose (∗∗*P* < 0.01), and LDH release declined (∗*P* < 0.05). It showed that tanshinone IIA inhibited radiation-induced cardiomyocyte injury while enhancing the proliferative capacity of the cells. When further administration of p38 MAPK agonist treatment, it was found that the cell clonogenic rate decreased significantly (∗∗∗*P* < 0.001), and the LDH level also increased significantly (∗∗*P* < 0.01), suggesting that p38 MAPK aggravated the damage and inhibited cell proliferation.

### 3.2. Tanshinone IIA Can Reduce Radiation-Induced Oxidative Stress in Cardiomyocytes

**Figure 3:**
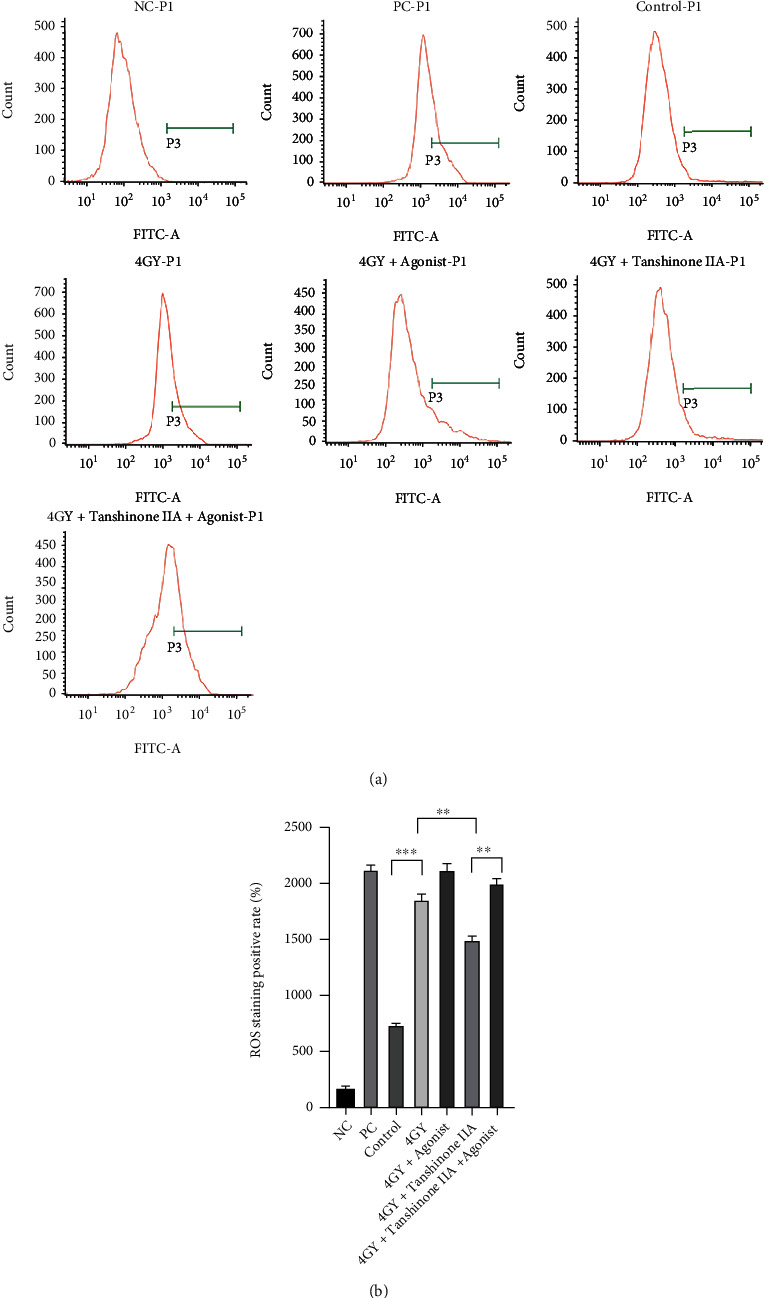
Effects of radiation and tanshinone IIA on oxidative stress in cardiomyocytes. (a) The ROS profile of each grouped cell was determined by flow cytometry. (b) The positive rates for ROS staining are expressed as mean ± SD. All results are presented as means ± SD (*n* = 3). ^∗∗∗^*P* < 0.001 and ^∗∗^*P* < 0.01. ROS: reactive oxygen species; NC: negative control; PC: positive control.

To explore whether tanshinone IIA has the ability to exert antioxidant activity, we performed ROS and SOD activity measurements, as shown in Figures 2(b) and 3. The experimental results showed that ROS increased in the H9c2 cells (∗∗∗*P* < 0.001), while SOD decreased in the 4 Gy group (∗∗*P* < 0.01), while LDH increased in the same way (∗∗∗*P* < 0.001). After treatment with tanshinone IIA, the antioxidant capacity of the cells clearly recovered. Tanshinone IIA increased the level of SOD (∗∗*P* < 0.01); meanwhile, both ROS (∗∗*P* < 0.01) and LDH (∗*P* < 0.05) decreased. When further treated with p38 MAPK agonists, LDH (∗∗*P* < 0.01), ROS (∗∗*P* < 0.01), and SOD (∗∗∗*P* < 0.001) all rose significantly, suggesting that p38 MAPK makes cellular oxidation and oxygen resistance become quite active, but at the same time, cellular damage is also aggravated.

### 3.3. Tanshinone IIA Can Decrease the Apoptosis of Cardiomyocytes Caused by Radiation

**Figure 4:**
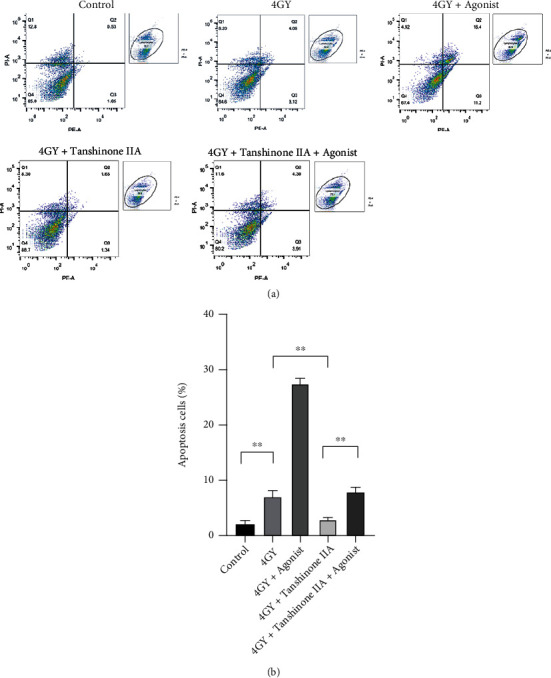
Effects of radiation and tanshinone IIA on cardiomyocyte apoptosis. (a) Flow cytometry was used to detect apoptosis in each group. (b) The results are expressed as mean ± SD (*n* = 3). ^∗∗^*P* < 0.01.

**Figure 5:**
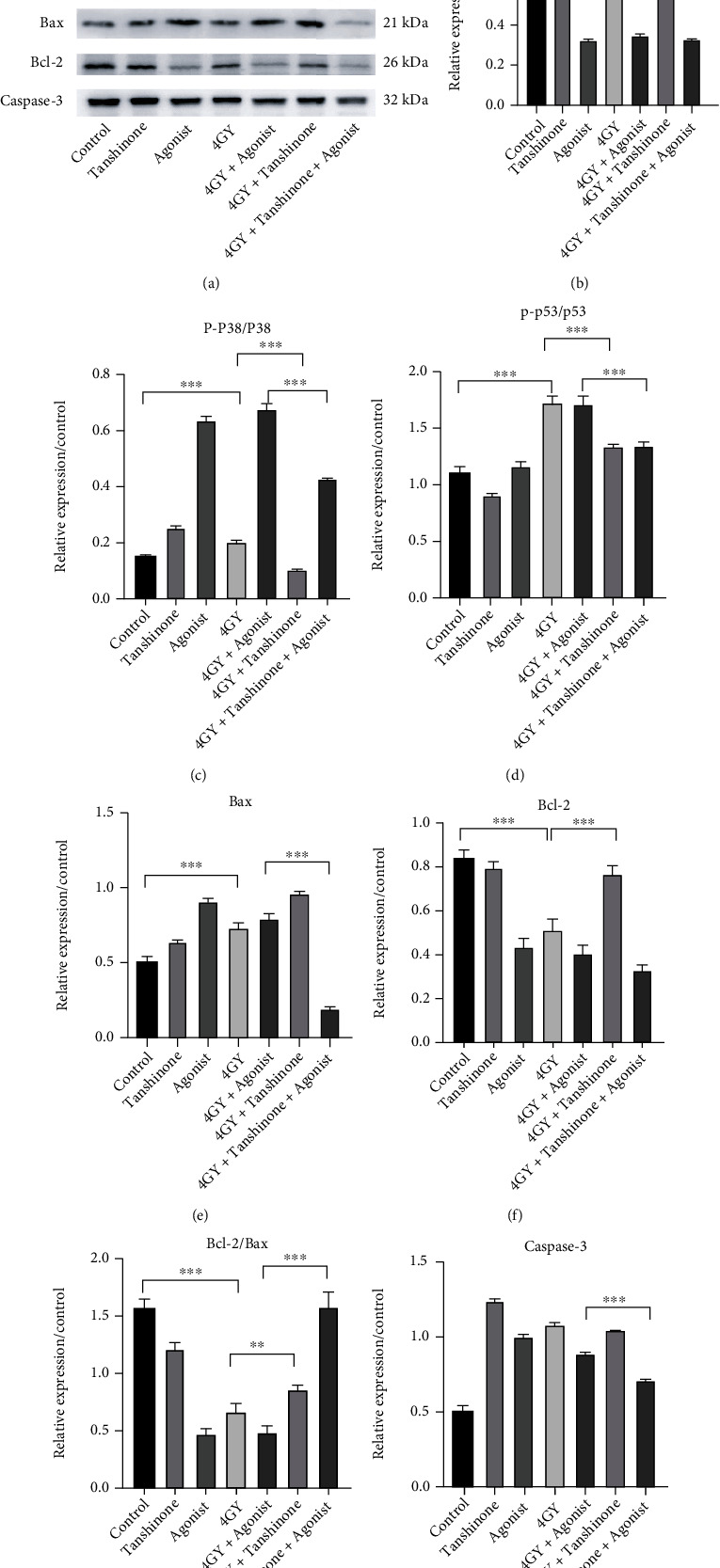
Expression of p38/p53 pathway related and apoptotic proteins. (a) It is a typical image of western blot. (b) P38 expression levels. (c) Ratio of p-p38/p38. (d) Ratio of p-p53/p53. (e) Bax expression levels. (f) Bcl-2 expression levels. (g) Ratio of Bcl-2/Bax. (h) Caspase-3 expression levels. GAPDH was used as reference. The results are expressed as mean ± SD. ^∗∗∗^*P* < 0.001 and ^∗∗^*P* < 0.01.

The cell apoptosis assay showed that tanshinone IIA inhibited radiation-induced cardiomyocyte apoptosis (∗∗*P* < 0.01). However, after the use of p38 MAPK agonists, the rate of apoptosis was again significantly increased (∗∗∗*P* < 0.001). According to the results of western blot analysis, after 4 Gy irradiation, Bax expression was elevated in cardiomyocytes, and there was a significant decrease in Bcl-2 and Bcl-2/Bax levels (∗∗∗*P* < 0.001). When it was found after tanshinone IIA treatment was administered, the Bcl-2/Bax ratio was elevated (∗∗*P* < 0.01). It showed that tanshinone IIA inhibited cell apoptosis. Finally, Bcl-2/Bax was elevated in the 4 Gy+tanshinone+agonist group (∗∗∗*P* < 0.001). The above results are shown in Figures 4 and 5.

### 3.4. Tanshinone IIA Downregulated Radiation-Induced p38/p53 Pathway Overexpression in Cardiomyocytes

We found that phosphorylation of p53 and p38 was overexpressed in cardiomyocytes after radiation, as compared to the control group (∗∗∗*P* < 0.001). It showed that the p38/p53 pathway was activated in radiation injury. After administration of tanshinone IIA, both p38 and p53 phosphorylation levels were downregulated, suggesting that tanshinone IIA has a regulatory effect on the p38/p53 pathway in radiation injury (∗∗∗*P* < 0.001). Comparing the 4 Gy+agonist group and 4 Gy+tanshinone IIA+agonist group revealed that tanshinone IIA could suppress the expression of p38/p53 activated by agonists (∗∗∗*P* < 0.001). The above results are shown in Figure 5.

## 4. Discussion

The thoracic tumor that has received radiotherapy can cause RIHD. At present, the research direction mainly focuses on inflammatory response, oxidative stress, and apoptosis [16, 17]. LDH is regarded as an indicator of cell damage. We found that in the present study, when tanshinone IIA treatment was administered to radiation injured cardiomyocytes, the cell clonogenic capacity was obviously restored, and the level of LDH decreased. This indicated that radiation-induced cardiomyocyte injury was reduced by tanshinone IIA, and the viability of the cells was restored.

For cells, antioxidation is an important self-protective mechanism that cells are able to utilize to resist damage caused by oxidants and/or electrophiles. There are many antioxidant proteins that counter the intracellular ROS, and these enzymes include thioredoxin reductase (TrxR), superoxide dismutase (SOD), catalase (CAT), and thioredoxin (TRX), among others [18]. SOD is a crucial antioxidant enzyme that guards against oxidative damage as organisms age. Therefore, the activation of intracellular SOD is crucial for the control of intracellular ROS. At present, many studies have found that radiation injury can cause abnormal elevation of ROS, which was similarly confirmed in this study, while the SOD can protect cells from oxidative stress [19, 20]. After tanshinone IIA administration, ROS decreased in cardiomyocytes, accompanied by SOD elevation, illustrating the elevation of cellular antioxidant capacity. Tanshinone IIA protected against radiation-induced cardiomyocyte injury in all of the above studies.

p38 MAPK plays important roles in cell proliferation, differentiation, inflammation, and apoptosis [21, 22]. Previous studies have shown that p38 can phosphorylate antiapoptotic proteins such as Bcl-2, MCL1, and Bclxl, thereby allowing these proteins to be degraded [23–26]. Meanwhile, p38 can phosphorylate Bax, making Bax unable to bind to Bcl-2 [27]. All the above studies can confirm the important role played by p38 in the process of apoptosis. p53 can be activated upon phosphorylation by p38 at site 46, and subsequently, another proapoptotic protein, PUMA, is activated, thereby exerting a proapoptotic effect [28–30]. In the present study, the p38/p53 pathway was activated in cardiomyocytes after radiation, whereas Bax expression increased and Bcl-2 decreased, which illustrated that p38/p53 played a role in apoptosis. When tanshinone IIA was administered, the p38/p53 expression decreased, and Bcl-2/Bax increased, and the apoptosis rate decreased. Similarly, comparing the 4 Gy+agonist group and the 4 Gy+tanshinone IIA+agonist group, we found that p38/p53 expression decreased and Bcl-2/Bax increased after administration of tanshinone IIA, which was consistent with the results of apoptosis experiment. We proved that the p38/p53 pathway was downregulated by tanshinone IIA in protecting radiation-induced myocardial injury.

In previous studies, tanshinone IIA has numerous protective effects on the myocardium. For example, tanshinone IIA can attenuate ventricular remodeling in rats with heart failure [31]. Tanshinone IIA is able to protect cardiac function by regulating angiogenesis in mice with myocardial ischemia [32]. Combined with previous studies by our group, tanshinone IIA could protect cardiomyocytes by reducing radiation-induced apoptosis through downregulation of the p38/p53 pathway. And it is predicted that p38 MAPK can be targeted for inhibition as a therapeutic approach to reduce radiation injury and provide a novel strategy to prevent and treat RIHD.

## Data Availability

All data generated or analyzed during this study are included in this published article.

## Abbreviations

7-AAD: 7-Aminoactinomycin D
ANOVA: Analysis of variance
Bax: B-cell lymphoma 2-associated X protein
BCA: Bicinchoninic acid
Bcl: B-cell lymphoma
Caspase: Cysteinyl aspartate specific proteinase
DCFH-DA: 2,7-Dichlorodi-hydrofluorescein diacetate
DMEM: Dulbecco's modified eagle medium
DNA: Deoxyribonucleic acid
GAPDH: Glyceraldehyde-3-phosphate dehydrogenase
LDH: Lactate dehydrogenase
MAPK: Mitogen-activated protein kinases
MCL1: Myeloid cell leukemia 1
PE: Phycoerythrin
PUMA: P53 upregulated modulator of apoptosis
RIHD: Radiation-induced heart disease
ROS: Reactive oxygen species
SOD: Superoxide dismutase
SPSS: Statistical Product Service Solutions.
